# The Arabidopsis Circadian Clock and Metabolic Energy: A Question of Time

**DOI:** 10.3389/fpls.2021.804468

**Published:** 2021-12-09

**Authors:** Luis Cervela-Cardona, Benjamin Alary, Paloma Mas

**Affiliations:** ^1^Centre for Research in Agricultural Genomics, CSIC-IRTA-Universidad Autónoma de Barcelona (UAB)-UB, Barcelona, Spain; ^2^Consejo Superior de Investigaciones Científicas, Barcelona, Spain

**Keywords:** circadian clock, metabolism, mitochondria, ATP, *Arabidopsis thaliana*

## Abstract

A fundamental principle shared by all organisms is the metabolic conversion of nutrients into energy for cellular processes and structural building blocks. A highly precise spatiotemporal programming is required to couple metabolic capacity with energy allocation. Cellular metabolism is also able to adapt to the external time, and the mechanisms governing such an adaptation rely on the circadian clock. Virtually all photosensitive organisms have evolved a self-sustained timekeeping mechanism or circadian clock that anticipates and responds to the 24-h environmental changes that occur during the day and night cycle. This endogenous timing mechanism works in resonance with the environment to control growth, development, responses to stress, and also metabolism. Here, we briefly describe the prevalent role for the circadian clock controlling the timing of mitochondrial activity and cellular energy in *Arabidopsis thaliana*. Evidence that metabolic signals can in turn feedback to the clock place the spotlight onto the molecular mechanisms and components linking the circadian function with metabolic homeostasis and energy.

## The Arabidopsis Circadian Clock: A Brief Overview

Multiple biological processes display a rhythmic oscillation with a period of approximately 24-h. The rhythms are sustained under constant environmental conditions indicating that are generated by a self-sustained timing mechanism or circadian clock. The core of the clock generates circadian rhythms in output processes through the daily and seasonal synchronization, orchestrated largely by changes in light and temperature (Young and Kay, 2001). Many of the mechanistic insights of the circadian clock function in plants derive from studies in *Arabidopsis thaliana* (Sanchez and Kay, 2016; McClung, 2019; Nakamichi, 2020). The number of clock components at the core of the oscillator has been considerably expanded over the last years. Increasing evidence also highlight a complex array of regulatory mechanisms that contribute to the rhythmic clock (Seo and Mas, 2014; Mateos et al., 2018; Chen and Mas, 2019; Nakamichi, 2020).

In broad outline, the current view of the circadian network includes a plethora of clock repressors that are expressed and function at specific times during the day and night. Clock repressors include the morning-expressed MYB transcription factors CIRCADIAN CLOCK ASSOCIATED1 (CCA1) and LATE ELONGATED HYPOCOTYL (LHY), as well as the members of the pseudo-response regulator (PRR) protein family PRR9 and PRR7 (Sanchez and Kay, 2016; McClung, 2019; Nakamichi, 2020). Other members of the PRR family such as PRR5 and TIMING OF CAB EXPRESSION1 (TOC1/PRR1, herein referred as TOC1) are expressed later during the day or close to dusk, respectively (Sanchez and Kay, 2016; McClung, 2019; Nakamichi, 2020). The Evening complex (EC) composed of ELF3 (EARLY FLOWERING 3, ELF4, and LUX/PCL1 (LUX ARRHYTHMO/PHYTOCLOCK1) functions in the evening to repress the *PRR* morning genes (Sanchez and Kay, 2016; McClung, 2019; Nakamichi, 2020). These repressors configure a complicated regulatory network, which is complemented by activating functions such as the rhythmic changes in chromatin marks (Chen and Mas, 2019) or clock components including LWD1 and 2 (LIGHT-REGULATED WD1 and 2) (Wu et al., 2008, 2016; Wang et al., 2011), or members of the RVE (REVEILLE) protein family (Farinas and Mas, 2011; Rawat et al., 2011; Hsu et al., 2013; Ma et al., 2018; Shalit-Kaneh et al., 2018), which directly interact with the members of the clock-related factors LNKs (NIGHT LIGHT-INDUCIBLE AND CLOCK-REGULATED GENES) to recruit the transcriptional machinery to activate clock gene expression (Ma et al., 2018).

The importance of the circadian function in Arabidopsis is reflected in the ample array of processes regulated by the clock. The processes pervade nearly every aspect of development, growth, or responses to biotic and abiotic stresses (Kinmonth-Schultz et al., 2013; Greenham and McClung, 2015; Sanchez and Kay, 2016; Mora-García et al., 2017). The circadian clock also intersects with many relevant cellular processes and pathways including among many others, aging (Kim et al., 2016), or the cell cycle (Fung-Uceda et al., 2018). Plants as other organisms, need to precisely control of the timing of energy production and partitioning, enabling them to produce enough energy to cover for the cellular energetic demands.

## Cellular Energy and Mitochondrial Activity in Arabidopsis

Adenosine triphosphate (ATP) is a key energy carrier molecule necessary for basic cellular functions in nearly all eukaryotic cells (Millar et al., 2011; Herst et al., 2017). Generation of ATP in mitochondria by oxidative phosphorylation relies on the transfer of electrons from reducing equivalents into oxygen (Braun, 2020). The reducing equivalents (such as nicotinamide adenine dinucleotide, NADH, and flavin adenine dinucleotide, FADH2) are formed through the tricarboxylic acid (TCA) cycle by the oxidation of organic compounds (Fernie et al., 2004). Coupling the respiratory electron transfer chain with the electrochemical proton gradient across the inner mitochondrial membrane is used by the ATP synthase complex to phosphorylate adenosine diphosphate (ADP), resulting in ATP to be used by the cell (Ishizaki et al., 2006). Additional non-phosphorylating by-passes [non-energy conserving electron transfer from cytoplasmic and matrix NAD(P)H to ubiquinone] are present in plants including alternative oxidase (AOX), NAD(P)H dehydrogenases, and uncoupling proteins (U) (Millar et al., 2011). When the canonical electron transfer chain is compromised, this alternative pathway allows the electron flux from NAD(P)H and succinate to oxygen without ATP synthesis, aiding the cell to cope with oxidative stress (Millar et al., 2011).

**FIGURE 1:**
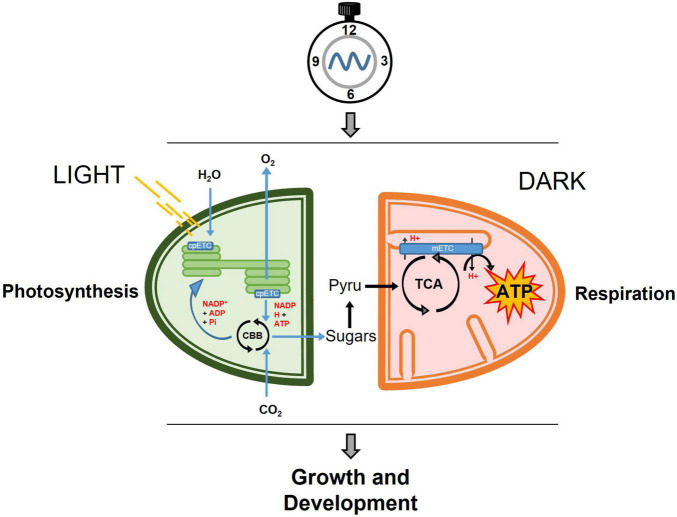
Simplified view of the temporal partitioning by the circadian clock of central metabolism in photosynthetic cells. During the day, carbon is fixed through the photosynthetic reactions in the chloroplast and exported in the form of soluble sugars to the cytosol where it will be converted into pyruvate. In the dark, pyruvate feeds the mitochondrial respiration through the TCA cycle, and subsequently the mitochondrial mETC to generate ATP, which will be used for growth and development. cpETC, chloroplastic electron transport chain; CBB, Calvin-Benson-Bassham cycle; Pyru, Pyruvate; TCA, Tricarboxylic Acid Cycle; mETC, mitochondrial electron transport chain; ATP, adenosine triphosphate.

In plants, mitochondrial and chloroplast activities are closely connected (Millar et al., 2011; Nunes-Nesi et al., 2011; Schertl and Braun, 2014). Both organelles behold electron transport chains, which are responsible for the generation (chloroplasts) or utilization (mitochondria) of reducing equivalents, translocation of protons and the creation of the proton gradient as the boosting force for ATP synthesis. These mitochondrial and chloroplastic metabolic reactions must be precisely coordinated in synchronization not only with the cellular status but also with the environmental conditions (Figure 1). Indeed, throughout the day, plants assimilate carbon in form of soluble sugars for immediate export and starch granules to be storage in the chloroplasts. Upon darkness, the stored starch is converted into glucose and maltose that are then exported to the cytosol to supply the energetic demands in form of sucrose. The generated sucrose and sucrose derivatives will be then converted by glycolysis into pyruvate, which will be oxidized through the TCA cycle.

Consistent with their primary function within the cell, both organelles show high metabolic plasticity. During the day, the TCA cycle intermediates are required for nitrogen assimilation in chloroplasts such that the TCA cycle shifts into a non-cyclic mode, affecting the formation of reducing equivalents (Sweetlove et al., 2010). The organelles are also able to trigger alternative metabolic routes to keep a quasi-normal metabolic pace bypassing the predominant oxidative pathways. For instance, under energy limitation conditions such as extended darkness, short period or drought, plant mitochondria can completely oxidize the Branched-Chain Amino Acids (BCAA) producing high amounts of ATP necessary for the plant to survive the metabolic stressful conditions (Pedrotti et al., 2018).

The bulk of ATP generation by plant mitochondria mostly occurs during the night. However, ATP produced by mitochondria at day time is important for other cellular processes such as sucrose biosynthesis and photorespiration (Bauwe et al., 2010). Photorespiration (also known as oxidative photosynthetic carbon cycle) relies on the uptake of molecular oxygen concomitant with the release of carbon dioxide. The gas exchange is similar to “dark” respiration, but the photorespiratory oxygen consumption occurs in chloroplasts, whereas the carbon dioxide is liberated in the mitochondria. The alternative NAD(P)H dehydrogenases contribute to the NADH re-oxidation capacities in mitochondria required in photorespiration (Bauwe et al., 2010). Also, the alternative NAD(P)H dehydrogenases and the AOX, contribute to the re-oxidization of the excess of reducing equivalents formed by the light reaction of photosynthesis (Braun, 2020).

In this review, we provide a glimpse of some of the main studies reporting the partitioning of cellular energy by the circadian clock in *A. thaliana*, with particular emphasis on mitochondrial function. Examples showing the feedback of metabolic signals controlling the clock are also briefly mentioned. This review does not attempt to comprehensively describe every study but rather to highlight the main findings connecting the circadian clock with the energetic balance in Arabidopsis. The readers are encouraged to consult excellent reviews focusing on the connection between the circadian clock and plant metabolism (e.g., Sanchez and Kay, 2016; Mora-García et al., 2017).

## Diel and Circadian Regulation of Mitochondrial Activity

**FIGURE 2:**
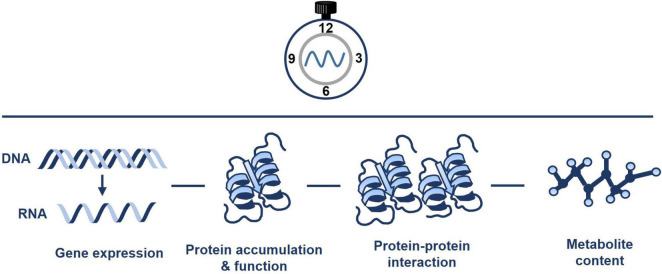
Pervasive diel and circadian oscillation of mitochondrial activity. The circadian clock regulates the timing of mitochondrial activity. Connections of the clock with mitochondria include the regulation of gene expression, protein accumulation and function, protein-protein interaction and metabolite content. Please refer to the text for further details.

The circadian clock controls the rhythms of many different metabolic pathways and organelles (Sanchez and Kay, 2016). Mitochondria are not an exception, and rhythmic trends of transcripts, proteins, metabolites, and ATP content have been documented (Figure 2). For instance, Lee et al. (2010) performed quantitative analysis of mitochondrial proteins over a diurnal cycle. The studies were complemented with analysis of enzyme activities and substrate-dependent respiratory processes in isolated mitochondria (Lee et al., 2010). The authors identified about 55 mitochondrial protein spots that dynamically changed over the diurnal cycle. The analyses also showed the diurnal changes in mitochondrial activity driving the TCA cycle and fluctuations associated with nitrogen and sulfur metabolism, and antioxidant defense. Additional studies integrating transcripts, metabolite and enzyme activity profiling during diurnal cycles in Arabidopsis rosettes also reported that the diurnal changes in metabolism-related transcripts generate nearly stable metabolites, and suggested that metabolites might modulate gene expression (Gibon et al., 2006). Altogether, the studies suggest a timely controlled adjustment of the mitochondrial respiratory capacity and metabolism to meet the energetic requirements over the diurnal cycle.

An specific example of rhythmic oscillation includes the *NAD-DEPENDENT MALIC ENZYME* (*NAD-ME*) genes, which encode members of the family of malic enzymes involved in metabolite flux through the TCA cycle (Tronconi et al., 2008). The *NAD-ME* gene expression and protein accumulation diurnally oscillate with peak abundance during the night. Metabolic analyses of *NAD-ME* loss-of-function mutants showed that these proteins might be important in the control of nocturnal metabolism, including the regulation of TCA intermediates, glucosinolates, cell wall components, isoprenoids, fatty acids, and plant immunity phytochemicals (Tronconi et al., 2008; Francisco et al., 2021). Together, the data suggest that NAD-ME may act as a key factor involved in the coordination of primary and secondary metabolism in a time-dependent manner. The expression of genes encoding other components of the TCA cycle for example ISOCITRATE DEHYDROGENASE (Gibon et al., 2006) or FUMARASE 2 (Gibon et al., 2006; Cervela-Cardona et al., 2021) was also shown to be diurnally or circadianly-regulated.

Light is intimately connected with the circadian function. A recent study has focused on identifying the dynamic changes in protein-protein interactions within the TCA cycle, the mitochondrial glycine metabolism, and the mitochondria respiratory pathway in response to light (Rugen et al., 2021). By using a differential complexome profiling strategy, the authors assayed protein-protein interactions at two time points: before the end of the day and before the end of the night. Comparative studies showed the formation of high molecular mass protein complexes in the mitochondrial matrix during the day, which differed from the ones observed at the end of the night. The mitochondrial complexes I and III, and the ATP synthase complex also showed altered abundance between the two conditions. The results thus pave the wave for a more comprehensive understanding of the mitochondria function though the dynamic regulation of protein-protein interactions during the day and night.

As mentioned above, the alteration of mitochondria activity or energy-deprivation conditions trigger the use of alternative respiratory pathways such as the catabolism of Branched-Chain Amino Acids (BCAA) (Pedrotti et al., 2018). Assessment of the rhythmic expression of *BCAA* genes with transcript co-expression analyses revealed positive correlations among *BCAA* catabolism genes and stress, development, diurnal/circadian, and light datasets (Peng et al., 2015). Transcript abundance was found to be reduced during the day and increased during the night. This oscillation was in agreement with a previous study showing that free *BCAA* abundance rhythmically oscillates with a peak at the end of the day (Gibon et al., 2006). Some of the *BCCA* transcripts also oscillated under constant light conditions, indicating a regulation by the circadian clock. Consistently, the expression of *BCAA* genes was altered in TOC1 miss-expressing plants (Cervela-Cardona et al., 2021), which suggest a redirected proteolytic metabolism due to the altered energy status in these plants.

The clock is also linked with photorespiration, which involves three different organelles: the mitochondrion, the chloroplast, and the peroxisome. Proper regulation of photorespiration requires a complex coordination of the expression of nuclear genes encoding proteins targeted to the three different organelles. Early studies showed that the Arabidopsis circadian clock controlled the rhythmic expression of genes encoding the chloroplastic Rubisco small subunit and Rubisco activase as well as the peroxisomal catalase, all components of the photorespiratory pathway (Pilgrim and McClung, 1993; Zhong et al., 1994; Zhong and McClung, 1996). Characterization of the Arabidopsis serine hydroxymethyltransferase (SHM) genes encoding the mitochondrial matrix components involved in photorespiration showed that they also exhibited circadian oscillations that were in phase with those described for other photorespiratory genes (McClung et al., 2000).

Consistent with the important role for the circadian clock coordinating the timing of the photorespiration, glycine, a major substrate for mitochondrial photorespiration (Lernmark et al., 1991) is altered in TOC1 miss-expressing plants (Cervela-Cardona et al., 2021). Proper expression and function of TOC1 might be thus important in the regulation of photorespiration (Nunes-Nesi et al., 2010). As fumarate content is also affected by miss-expression of TOC1 (see below), it is possible that the changes in fumarate alter photorespiration and nitrogen metabolism during the day, leading to a depletion (in TOC1-ox) or accumulation (in *toc1-2*) of glycine (Cervela-Cardona et al., 2021).

Most of the studies in the laboratory are performed using chambers with environmentally-controlled conditions. However, these conditions usually differ or do not realistically recapitulate the fluctuations occurring under natural environments. Comparative analyses using plants grown under artificial light and sunlight conditions showed that the metabolism of organic acids and amino acids was less robust than that of starch turnover (Annunziata et al., 2017, 2018). These, and some other reports (Nagano et al., 2012; Matsuzaki et al., 2015; Song et al., 2018) highlight the necessity of growing plants under more natural field conditions. Additional studies incorporating coordinated changes in irradiance and temperature also showed an altered amplitude and a delayed peak phase of expression of circadian clock genes expressed around dawn such as *LHY*, *CCA1*, and *PRR9* (Annunziata et al., 2017, 2018). The authors proposed that the changes in phase might delay the clock activity until the temperature rises to allow metabolic activities such as photosynthesis to commence.

## Components and Mechanisms Connecting the Circadian Clock With Metabolic Energy

One key approach to examine the circadian regulation of metabolism relies on the use of relevant clock mutants and over-expressing lines in order to examine changes in metabolism-related gene expression, protein abundance, enzyme activity, or metabolite content. Several studies have used this reverse genetic approach. One prime example includes the use of the *prr975* triple mutant (Fukushima et al., 2009). Metabolite profiling showed that the *prr975* mutant plants displayed a significant increase of many intermediates of the TCA cycle. Combining transcriptomics and metabolomics uncovered the role of PRR9, PRR7, and PRR5 in the regulation of the biosynthetic pathways of chlorophyll, carotenoid, abscisic acid, and α-tocopherol (Fukushima et al., 2009). Thus, mitochondria and chloroplast homeostasis are direct circadian clock outputs and proper expression and function of these PRR components is important to sustain the metabolic homeostasis.

Another example includes the clock component TIME FOR COFFEE (TIC), which contributes to clock resetting at dawn (Hall et al., 2003; Ding et al., 2007). Analyses with the loss of function mutant showed major changes in the expression of genes related to various pathways including metabolism (Sanchez-Villarreal et al., 2013). Consistently, *tic* mutant plants showed increased glutathione, and readjustments of amino acids and polyamine pools, which correlated with phenotypes such as excess of starch, altered soluble carbohydrate abundance, hypersensitivity to oxidative stress and resistance to drought. Comparative analyses of transcriptomic and metabolomic data confirmed a role for TIC regulating plant metabolism (Sanchez-Villarreal et al., 2013). Further studies also uncovered a genetic interaction between TIC and AKIN10, a catalytic subunit of the evolutionarily conserved key energy sensor sucrose non-fermenting 1 (Snf1)-related kinase 1 (SnRK1) (Shin et al., 2017). The study showed that inducible over-expression of *AKIN10* lengthened the circadian period and delayed the phase of the clock gene *GIGANTEA* (*GI*), and that this regulation required a functional TIC (Shin et al., 2017).

The analyses of two time points (end-of-day and end-of night) by RNA-sequencing and protein mass spectrometry with a battery of core clock mutants also revealed particular sets of mitochondrial-related genes and proteins miss-regulated for each core clock component (Graf et al., 2017). The comparative analyses also showed the lack of correlative changes in protein abundance and in the corresponding transcript in many instances. Transcripts and proteins with coordinated changes in abundance were enriched in carbohydrate- and cold-responsive genes, whereas genes encoding transcription factors, starch degradation enzymes, and protein kinases were found in all circadian clock mutants examined. The authors highlighted the importance of post-translational modifications and protein degradation to fully understand the physiological and metabolic processes controlled by the clock (Graf et al., 2017).

The use of mutants affecting clock genes expressed throughout the circadian cycle (dawn, morning, dusk, and evening) also uncovered the role of the circadian clock regulating starch synthesis and the accumulation of organic acids and amino acids in the light (Flis et al., 2019). The results indicated that dawn components positively contributed to the accumulation of amino acids, whereas the evening component likely activated starch accumulation but repressed sucrose recycling. Furthermore, the authors proposed that the circadian clock was able to compensate metabolism against the high load of carbon from photosynthesis. Thus, a complex array of clock outputs regulate the turnover of carbon and nitrogen reserves (Flis et al., 2019).

**FIGURE 3:**
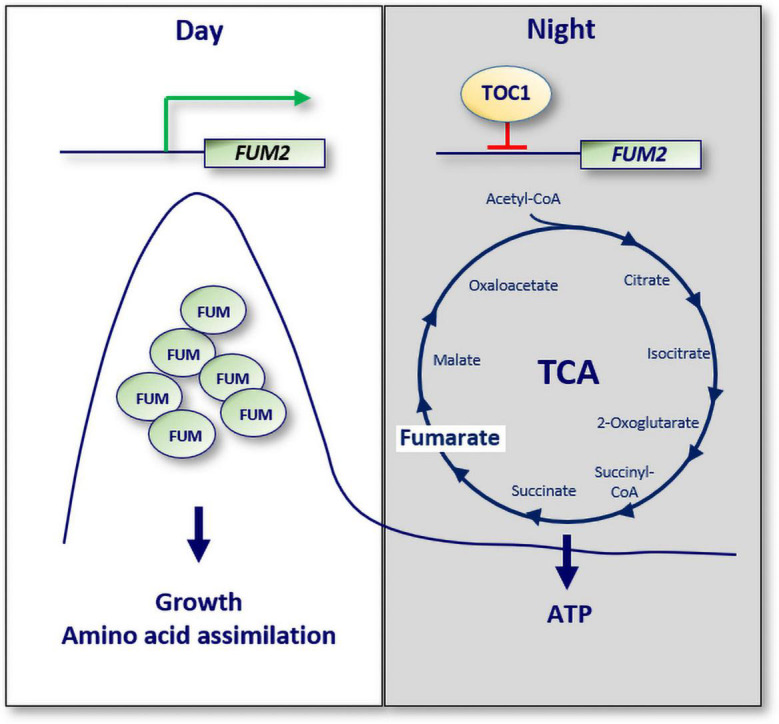
A molecular mechanism connecting the circadian clock with cellular ATP. The expression of the TCA cycle related gene known as *FUMARASE 2 (FUM2)* oscillates with a peak phase during the day. Fumarate accumulation is important for growth and amino acid assimilation. TOC1 binds to the promoter of *FUM2* to repress its expression at night. Consequently, the ATP/ADP ratio, a measure of energy homeostasis, is altered in TOC1 miss-expressing plants. White box: day; gray box: night; Green arrow: activation; red lines: repression.

A recent study has also provided a molecular mechanism connecting the circadian clock with mitochondria function (Cervela-Cardona et al., 2021). The study showed that TOC1 is important for sustaining the rhythms of sugars, amino acids and a number of TCA cycle intermediates. TOC1 also regulates the rhythms of ATP production by mitochondria. Consistently, miss-expression of TOC1 results in a variety of molecular and physiological phenotypes resembling energy-deprivation status. The regulation of metabolism by TOC1 relies on its binding to the promoter of the TCA-related gene *FUMARASE 2* (*FUM2*), which correlates with the repression of *FUM2* expression at night, and with reduced fumarate accumulation (Figure 3). The results thus provide a mechanistic explanation for the diurnal and circadian regulation of *FUM2* expression and fumarate accumulation (Chia et al., 2000; Pracharoenwattana et al., 2010; Cervela-Cardona et al., 2021). Altogether, the study suggests that TOC1 and its control of *FUMARASE 2* contribute to the regulation of energy homeostasis during the day and night.

Additional molecular connectors between mitochondria and the circadian clock include the TCP (TEOSINTE BRANCHED 1, CYCLOIDEA, and PCF) family of transcriptions factors, which regulate plant growth and development (Nicolas and Cubas, 2016). Promoter analyses identified site II regulatory elements [TGGGC(C/T)] that contribute to the transcriptional activation or repression of genes encoding mitochondria-related proteins (Giraud et al., 2010). TCP transcriptions factors not only bind to the site II elements but also interact with core clock components such as LHY and PRR5. Therefore, the TCP proteins connect the circadian clock with mitochondrial function through their binding to the site II elements to control gene expression and organelle protein abundances (Giraud et al., 2010).

## Metabolic Feedback to the Circadian Clock

Metabolites and metabolic changes in turn feedback to regulate the diurnal and circadian oscillation of gene expression. For instance, nitrogen (N) is an important nutrient and nitric oxide (NO) is used as metabolic signal that regulates gene expression in plants (Balotf et al., 2018). Gutiérrez et al. (2008) identified glutamate (Glu) or glutamine (Gln) as regulators of *CCA1* expression. The authors also found that CCA1 in turn contributed to the N-assimilatory pathway, providing a novel feedback mechanism by which N-assimilation functions as an input to the circadian clock, while in turn the clock regulates N-assimilation through CCA1 (Gutiérrez et al., 2008). These results were consistent with previous studies showing that the expression of two nitrate reductase genes, *NIAI* and *NIA2* (Cheng et al., 1988; Crawford et al., 1988) showed diurnal oscillations with peak abundance occurring shortly after dawn (Cheng et al., 1991; Pilgrim and McClung, 1993). The oscillations were sustained under constant light and under constant darkness indicating that rhythms were controlled by the circadian clock. The oscillation of gene expression was functionally relevant, as the circadian oscillations in *NIA2* expression correlated well with rhythms in nitrate reductase activity.

The use of the starchless phosphoglucomutase (*pgm*) mutant (Caspar et al., 1985) also uncovered that endogenous changes of sugars regulated around half of the circadian-controlled genes encoding putative transcription factors, and proteins involved in the regulation of DNA, RNA, protein synthesis, and degradation (Blasing et al., 2005). The analyses suggested that low abundance of sugars at the end of the night were responsible for the majority of the changes. Mathematical modeling also showed that the dynamic adjustments of the circadian clock to cellular sucrose modulated starch turnover, and thus, the circadian responses to sucrose signals contribute to carbon homeostasis and improved growth (Seki et al., 2017).

Sugar signals were also shown to entrain the clock through the morning-expressed clock component PRR7 (Haydon et al., 2013; Frank et al., 2018). Mathematical analyses and experimental validation also identified the clock component GIGANTEA (GI) as an important sensor necessary for the full response of the circadian clock to sucrose (Dalchau et al., 2011). A number of additional molecular components contributing to the adjustment of the circadian phase by sugars has been also identified. Examples include the TREHALOSE-6-PHOSPHATE SYNTHASE1 (TPS1) and the sugar-sensing kinase SnRK1 that controls the transcription factor BASIC LEUCINE ZIPPER63, which in turn regulates *PRR7* expression in response to sugars (Frank et al., 2018).

Another factor required for the circadian clock to respond to sucrose is SENSITIVE TO FREEZING 6 (SFR6) (Knight et al., 2008). The changes in amplitude and phase of clock-related genes induced by sucrose were found to be reduced in *sfr6* mutant plants. Sucrose can also impact the circadian oscillator by increasing superoxide abundance (Román et al., 2021). Indeed, not only superoxide-regulated transcripts are regulated by the circadian clock, but also the regulation of *TOC1* expression by sucrose in the evening requires superoxide. Thus, the authors provide evidence for a role for superoxide as part of the sugar signaling pathway regulating the rhythms of clock gene expression.

A recent study has also reported a mechanism contributing to the metabolic sugar input to the circadian clock (Shor et al., 2017). The study showed that light not only drives photosynthesis but also favors the phosphorylation and degradation of the PHYTOCHROME INTERACTING FACTORS (PIFs), the negative regulators of the phytochrome signaling (Paik et al., 2017). However, the sugars produced by photosynthesis function in an opposite way to light i.e., activate PIFs abundance and the binding to the promoters of *CCA1* and *LHY*, to activate their expression. Thus, the study uncovers a role of PIFs regulating sucrose signals to the oscillator (Shor et al., 2017).

Early studies also showed that cyclic adenosine diphosphate ribose (cADPR), driving the circadian oscillations of Ca^2+^ release, was able to regulate the oscillator gene expression (Dodd et al., 2007). Another recent study has also shown that the target of rapamycin (TOR) kinase, a central regulator of growth, contributes to the glucose- and nicotinamide-dependent regulation of circadian period by the clock (Zhang et al., 2019). The study proposes that nicotinamide regulates circadian period length by blocking the glucose-TOR energy signaling. Thus, the TOR kinase might function as an energy sensor for the timely coordination of the circadian clockwork and plant growth.

## Final Remarks

Altogether, these studies convey the intimate relationship between the Arabidopsis circadian clock, mitochondria activity, and the overall metabolic homeostasis of the cell. Neither chloroplasts are just sites for photosynthesis and deposition of storage materials, nor mitochondria are just the powerhouse of the cell. Chloroplasts aid on the compartmentalization of the intermediary metabolism of cells. Purine and pyrimidine synthesis, most amino acid biosynthesis, and all the fatty acid synthesis take place in chloroplasts. Mitochondria are also involved in subsidiary functions to the primary metabolism by participating in the photorespiration process, redox status and nitrogen assimilation. It would be thus interesting to identify the full contribution of the circadian clock in the regulation of intermediary metabolism and the other processes and pathways controlled by the plant metabolic organelles.

The range of energetic demands during different plant developmental stages and importantly, in different parts of the plant, calls for more specific studies focused on the metabolic status in particular cells, tissues, or organs at different time points of the plant life cycle. The need for a precise and timely coordination of photosynthesis, respiration, and photorespiration also raises the question about the role of the circadian clock controlling the temporal partitioning of the activity in mitochondria, chloroplasts, and peroxisomes in response to diurnal and seasonal changes. Identifying the molecular components and regulatory mechanisms for such a coordination lies as a challenge in front of us. Analyses of metabolic changes in response to external abiotic and biotic threats, and the importance of the circadian clock contributing to metabolic homeostasis under these conditions would be quite relevant, particularly in the context of the climate change. Studies expanding the connection of the circadian clock and metabolism in crops of agronomical importance will also provide useful tools for biotechnological manipulation of the circadian regulation of metabolism in order to improve crop fitness, growth and productivity in resonance with the environment.

## Author Contributions

All authors listed have made a substantial, direct, and intellectual contribution to the work, and approved it for publication.

## Conflict of Interest

The authors declare that the research was conducted in the absence of any commercial or financial relationships that could be construed as a potential conflict of interest.

## Publisher’s Note

All claims expressed in this article are solely those of the authors and do not necessarily represent those of their affiliated organizations, or those of the publisher, the editors and the reviewers. Any product that may be evaluated in this article, or claim that may be made by its manufacturer, is not guaranteed or endorsed by the publisher.
